# Differential arousal regulation by prokineticin 2 signaling in the nocturnal mouse and the diurnal monkey

**DOI:** 10.1186/s13041-016-0255-x

**Published:** 2016-08-18

**Authors:** Qun-Yong Zhou, Katherine J. Burton, Matthew L. Neal, Yu Qiao, Anumantha G. Kanthasamy, Yanjun Sun, Xiangmin Xu, Yuanye Ma, Xiaohan Li

**Affiliations:** 1 Department of Pharmacology, University of California, Irvine, CA USA; 2 Department of Biomedical Sciences, Iowa State University, Ames, IA USA; 3 Department of Neurosurgery, Kunming Medical University Hospital, Yunan, China; 4 Department of Anatomy and Neurobiology, University of California, Irvine, CA USA; 5 Institute of Primate Translational Medicine, Kunming University of Science and Technology, Yunnan, China

**Keywords:** Circadian clock output, Wakefulness, Sleep, Diurnal, Nocturnal, Prokineticin 2, Oscillation, Intrinsically photosensitive retinal ganglion cells, Suprachiasmatic nucleus, Superior colliculus

## Abstract

The temporal organization of activity/rest or sleep/wake rhythms for mammals is regulated by the interaction of light/dark cycle and circadian clocks. The neural and molecular mechanisms that confine the active phase to either day or night period for the diurnal and the nocturnal mammals are unclear. Here we report that prokineticin 2, previously shown as a circadian clock output molecule, is expressed in the intrinsically photosensitive retinal ganglion cells, and the expression of prokineticin 2 in the intrinsically photosensitive retinal ganglion cells is oscillatory in a clock-dependent manner. We further show that the prokineticin 2 signaling is required for the activity and arousal suppression by light in the mouse. Between the nocturnal mouse and the diurnal monkey, a signaling receptor for prokineticin 2 is differentially expressed in the retinorecipient suprachiasmatic nucleus and the superior colliculus, brain projection targets of the intrinsically photosensitive retinal ganglion cells. Blockade with a selective antagonist reveals the respectively inhibitory and stimulatory effect of prokineticin 2 signaling on the arousal levels for the nocturnal mouse and the diurnal monkey. Thus, the mammalian diurnality or nocturnality is likely determined by the differential signaling of prokineticin 2 from the intrinsically photosensitive retinal ganglion cells onto their retinorecipient brain targets.

## Introduction

The temporal organization of activity/rest or sleep/wake rhythms for mammals is regulated by the interaction of light/dark cycle and circadian clocks. Several lines of evidence indicate that the known master circadian clock, suprachiasmatic (SCN), operates quite similarly in nocturnal and diurnal mammals. The oscillations of clockwork genes, such as Bmal1, Per1, and Per2, are in the same phase in the SCN, regardless whether the mammals are diurnal or nocturnal [1, 2]. The firing rate and the glucose utilization of SCN neurons are also in the same phase for both the nocturnal and the diurnal mammals [3, 4]. The same phase oscillation has also been shown for the two SCN output molecules, vasopressin and prokineticin 2 (PK2) [5–7]. Therefore, the divergent mechanisms that confine the active phase to either day or night period for the diurnal and the nocturnal mammals have been postulated to lie downstream of the SCN clock [4, 8, 9]. However, no such divergent mechanism has been identified.

Besides modulating the activity/rest or the sleep/wake rhythms indirectly via its ability to phase shift and entrain the SCN circadian clock to the ambient light–dark cycle, light also exerts a direct effect on the activity or arousal levels. In the nocturnal animals, light strongly suppresses activity and induces sleep (photosomnolence) [10, 11]. In the diurnal mammals, such as monkeys and humans, light produces opposite effects of inducing the arousal or increasing the activity levels [12–14]. For the nocturnal animals, the direct light effect of activity suppression is commonly referred to as masking [10, 11, 15]. Light-induced activity suppression and circadian clock entrainment appears to utilize the identical photic input pathways from the retina. Both classical (rod/cone) photoreceptors and intrinsically photosensitive retinal ganglion cells (ipRGC), the retinal ganglion cells that express melanopsin (OPN4), participate in the masking and the circadian clock entrainment, as well as other non-image-forming visual responses such as the pupillary reflex [11, 16–19]. Masking is attenuated in Opn4-deficient mice [18], and is essentially abolished in the mice deficient in both Opn4 and rod photoreceptors [20]. Masking and circadian clock entrainment to light/dark cycle is completely abolished in the mice lacking the ipRGC generated by genetic or chemical ablation [21–25]. Thus, the ipRGC are the only channels that relay the photic information to brains for the masking and the circadian clock entrainment. The neural pathway of mediating the light masking downstream of the ipRGC is thought to act through the retinohypothalamic tract (RHT) projection to the SCN [9, 11], the same neural pathway that mediates the phase shifting and the entrainment of the SCN clock. The light masking was abolished with complete transection of RHT [26]. SCN lesion together with the loss of RHT eliminates the circadian rhythmicity as well as the light masking [27]. Transplantation of embryonic SCN to arrhythmic adults restores some locomotor rhythmicity without restoring the masking effect [28]. Thus, the ipRGC-SCN neural pathway appears to be critical for the light masking in the nocturnal animals.

In the current study, we show the oscillatory expression of PK2, previously demonstrated as a critical SCN output signal, in the ipRGC in a clock-dependent manner. We further show that PK2 signaling is required for the sustained light-induced activity suppression and sleep induction in mice. Blockade with PK2 antagonist demonstrated the opposite effects of the PK2 signaling on the arousal levels in the nocturnal mouse and the diurnal monkey. Together with the observed differential expression of a PK2 signaling receptor in the retinorecipient brain targets of the ipRGC between the nocturnal mouse and the diurnal monkey, the mammalian diurnal/nocturnal determination lies among the ipRGC-brain pathways, upstream of the SCN clock.

## Results

### PK2 signaling is required for the sustained light-induced suppression of the locomotor activity and the arousal in mice

**Fig. 1:**
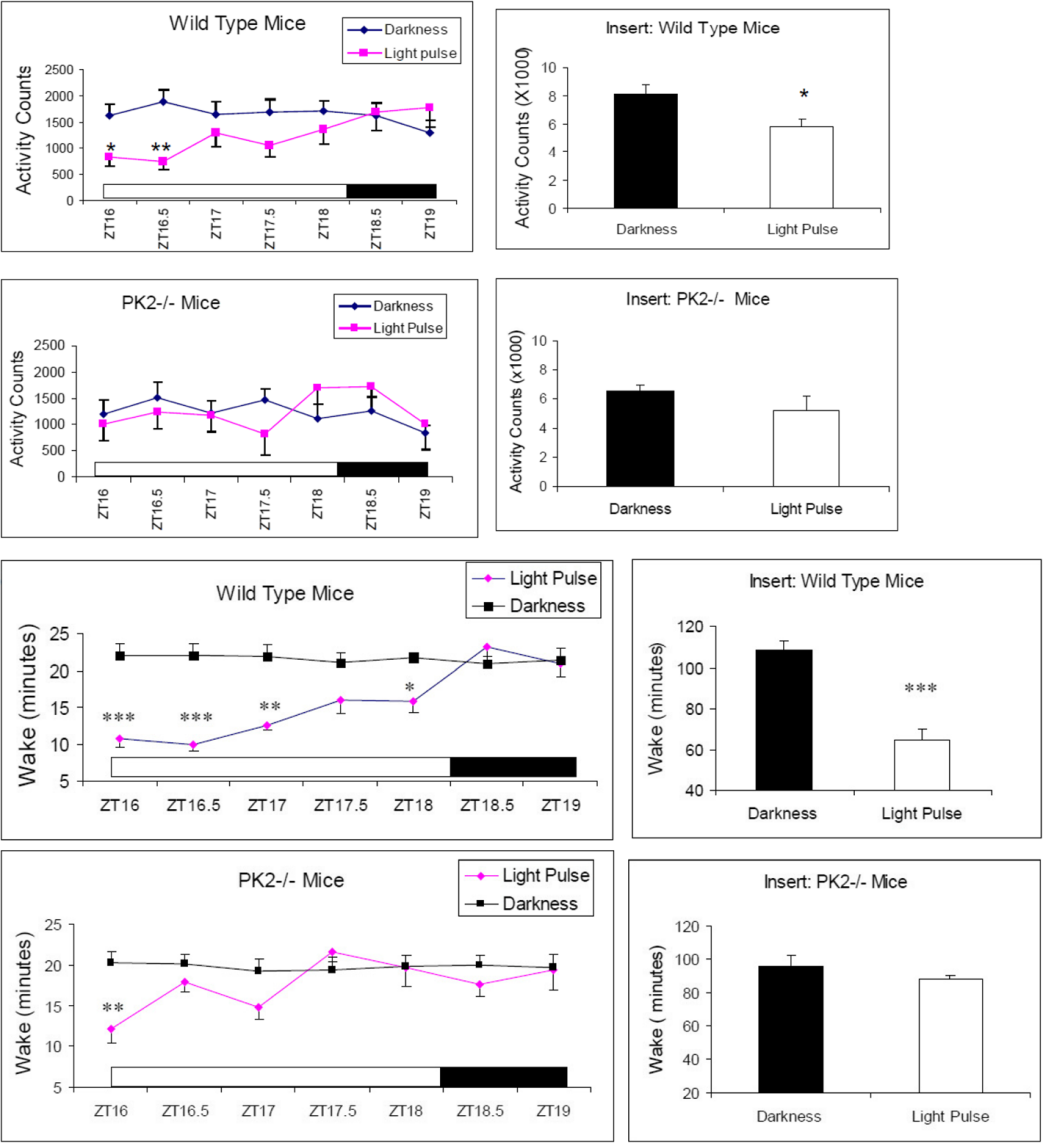
The effects of light pulses on the locomotor activities and the arousal levels. The white bar shows the light pulses (150 lux) administered to WT (*N* = 6) and PK2−/− mice (*N* = 7) during ZT16-ZT18.5 (2.5 h). The analysis bin sizes were 30 min, with values being means ± sem. **a**. Light pulses significantly inhibited the locomotor activity in the WT mice (*P* < 0.01, Two-way ANOVA, *, *P* < 0.05, **, *P* < 0.01 by Bonferroni’s post hoc test). The effect of light pulses on the locomotor activity of the PK2−/− mice was not significant (*P* > 0.05, Two-way ANOVA). The insert shows the locomotor activity in the entire 2.5 h (*, *P* < 0.05, paired t-test). **b**. Light pulses significantly deceased the wake time in the WT mice (*P* < 0.0001, Two-way ANOVA, *, *P* < 0.05, **, *P* < 0.01, ***, *P* < 0.001 by Bonferroni’s post hoc test). The inhibitory effect of light pulses on the arousal levels of the PK2−/− mice was only significant for the first 30 min (*P* < 0.05, Two-way ANOVA, **, *P* < 0.01, by Bonferroni’s post hoc test). The insert shows the wake minutes of the entire 2.5 h (***, *P* < 0.001, paired t-test). The inhibitory effect of light pulse on the arousal levels in the entire 2.5 h was not significant for the PK2−/− mice (*P* > 0.05, paired t-test)

Mice deficient in genes of either PK2 (PK2−/−) or its receptor (PKR2) had reduced circadian rhythms of locomotor activity under constant darkness condition [29, 30]. We observed that the PK2−/− mice also displayed increased daytime locomotor activity under light/dark (LD) cycle (data not shown), which is consistent with prior observation of increased wakefulness in the PK2−/− mice during light period [31]. These observations suggested that the light suppression effect is abnormal in the absence of PK2 signaling. We thus examined the suppression effect of light pulses on the locomotor activities and the arousal levels in the PK2−/− mice. When a light pulse (150 lux) was administrated to the wild type mice during the middle of dark period (ZT16-ZT18.5), the locomotor activities were significantly suppressed (Fig. 1a). In contrast, only marginal suppression of the locomotor activities by light pulses was observed for the PK2−/− mice (Fig. 1a and Insert). As expected, EEG/EMG recording revealed that light pulses suppressed wakefulness in the wild type mice (Fig. 1b and Insert). For the PK2−/− mice, the light suppression on the wakefulness was only significant for the first 30 min (Fig. 1b). Thus, light could still suppress the wakefulness in the PK2−/− mice, but the suppression effect was not maintained.

**Fig. 2:**
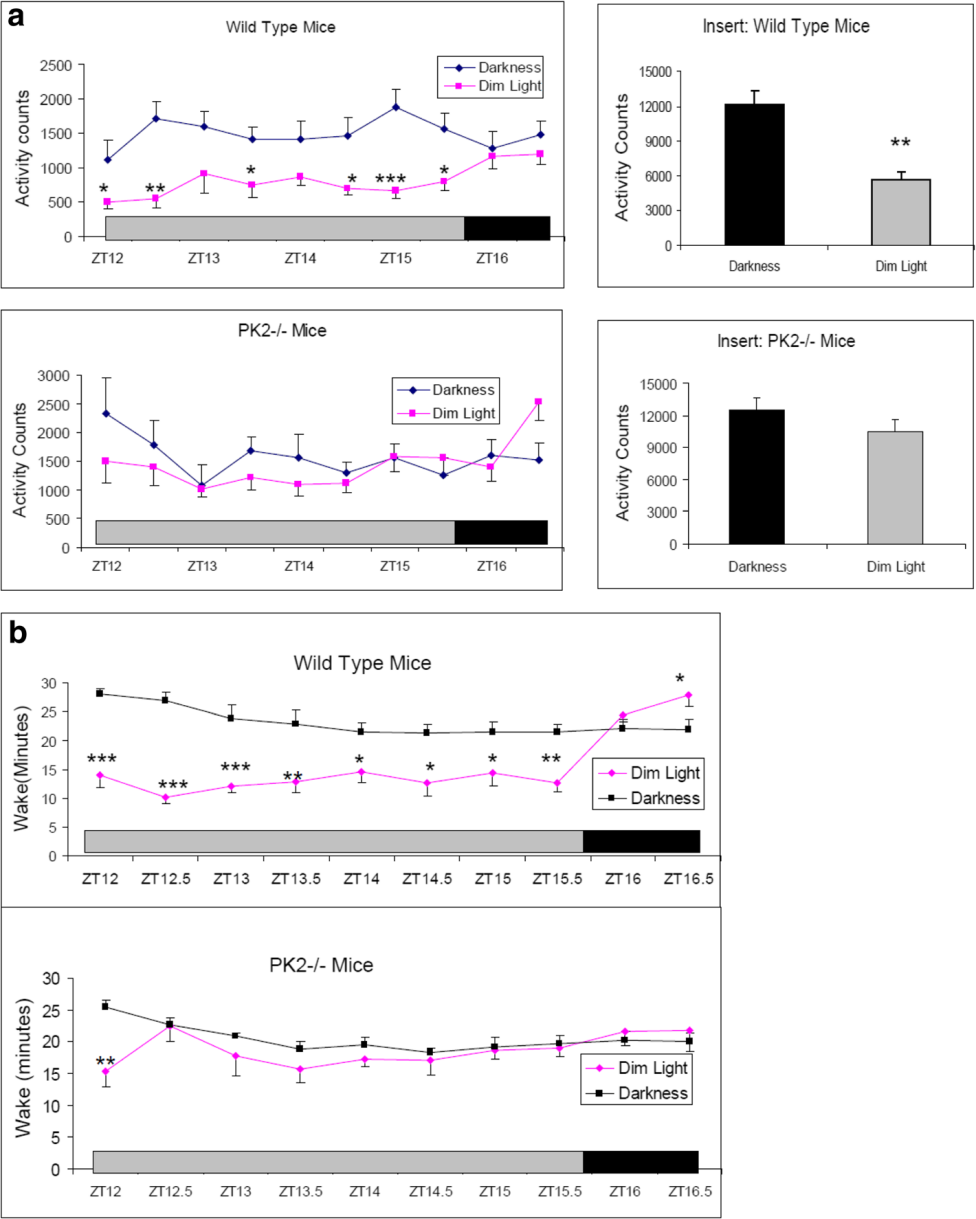
The effects of dim light on the locomotor activities and the arousal levels. The grey bar shows the four hours of dim light (30 lux) that was administered during ZT12-ZT16 to the WT (*N* = 6) and the PK2−/− mice (*N* = 7). **a**. Compared to darkness, dim light significantly decreased the locomotor activity in the WT mice (*P* < 0.0001, Two-way ANOVA, *, *P* < 0.05, **, *P* < 0.01, ***, *P* < 0.001 by Bonferroni’s post hoc test). The inhibitory effect of dim light on the locomotor activity was not significant for the PK2−/− mice (*P* > 0.05, Two-way ANOVA). The inserts show the locomotor activities in the entire four hours (**, *P* < 0.01, paired t-test). **b**. Compared to darkness, dim light significantly decreased the arousal levels in the WT mice (*P* < 0.0001, Two-way ANOVA, *, *P* < 0.05, **, *P* < 0.01, ***, *P* < 0.001, by Bonferroni’s post hoc test). The inhibitory effect of dim light on the arousal levels of the PK2−/− mice was only significant for the first 30 min (*P* < 0.05, Two-way ANOVA, **, *P* < 0.01, by Bonferroni’s post hoc test)

We further investigated the light suppression effect on the locomotor activities and the arousal levels with dim light. Just before the ending of the regular ~150 lux illumination condition at ZT12, the light intensity was dimmed to ~30 lux during the four hour period corresponding to ZT12-ZT16. This reduced illumination continuously suppressed the locomotor activities of the wild type mice (Fig. 2a). In contrast, the PK2−/− mice displayed quite robust locomotor activities in response to the light intensity reduction, achieving the activity levels quite comparable to that were under darkness (Fig. 2a). This observation indicated that, at the high homeostatic drive for the activities during late day, dim light was no longer able to markedly suppress the locomotor activities in the PK2−/− mice. EEG/EMG recording confirmed the corresponding arousal levels affected by this dim light treatment. For the wild type mice, the time staying awake during these four hours of dim light was much more reduced than under darkness, revealing a strong sleep induction effect of the dim light (Fig. 2b). For the PK2−/− mice, the wakefulness time under dim light and under darkness were quite similar, indicating only marginal arousal inhibition by the dim light in the absence of PK2 signaling (Fig. 2b). Together, these results indicate that PK2 signaling is required for the sustained light-induced suppression of the locomotor activity and the arousal in mice.

### Clock-dependent oscillatory expression of PK2 in the intrinsically photosensitive retinal ganglion cells (ipRGC)

**Fig. 3:**
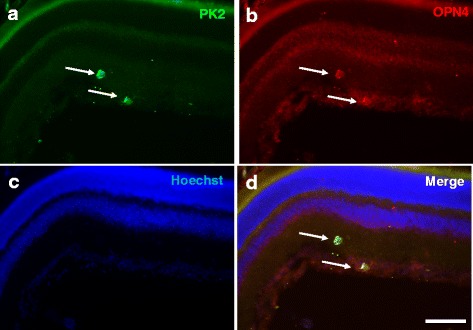
Coexpression of PK2 and OPN4 in retinal ganglion cells of the mouse retina. The PK2 immunostaining is shown as green (**a**) whereas the OPN4 fluorescent immunolabeling is shown in red (**b**). The nuclear counterstaining is shown as blue (**c**). Coexpression of PK2 and OPN4 in the retinal ganglion cells is apparent (**d**). Two cells strongly immunostained by PK2 and OPN4, indicated by arrows, are shown. Scale bar, 40 μm

**Fig. 4:**
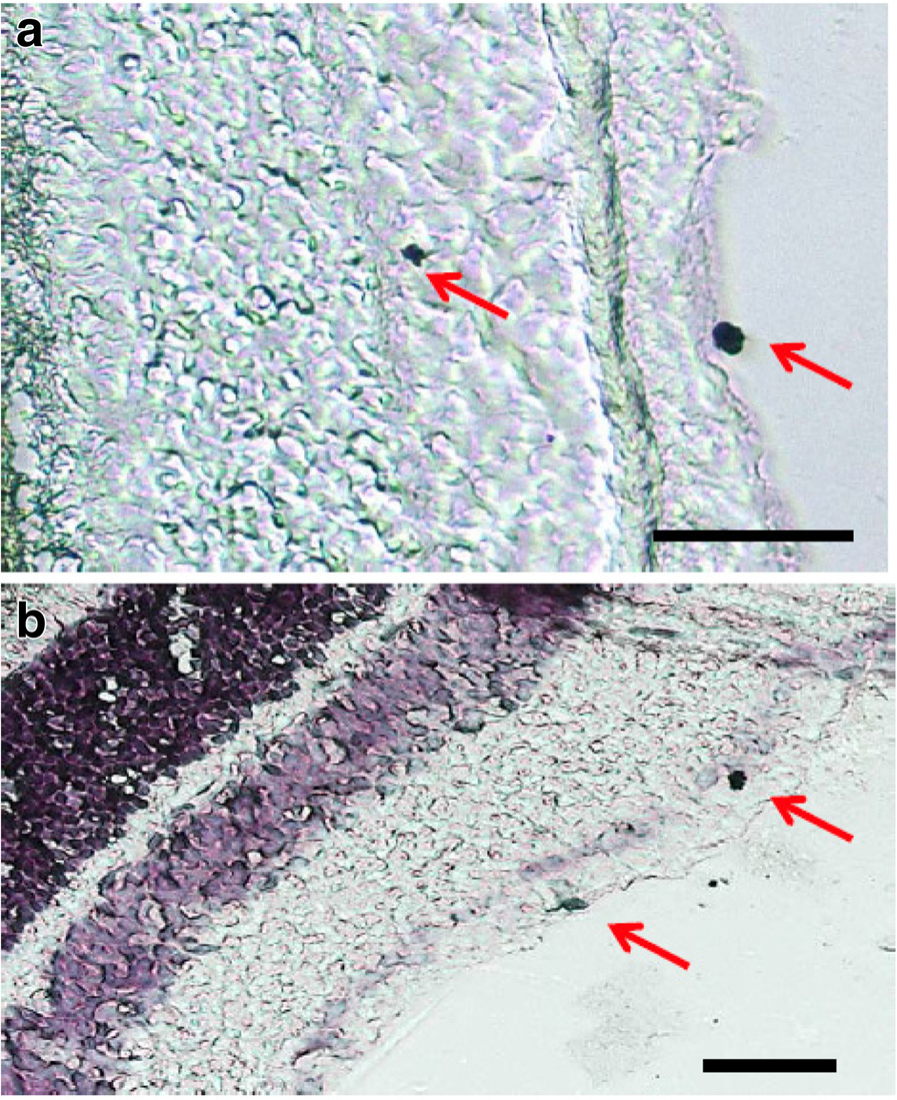
Expression of PK2 in the retinal ganglion cells of the mouse retina. PK2 immunolabeling was developed by DAB (3,3′-diaminobenzidine). **a**. Examples of two PK2-positive retinal ganglion cells, one nondisplaced and one displaced, are marked with arrows. **b**. PK2 DAB immunostaining with hematoxylin counterstaining. Examples of two PK2-positive retinal ganglion cells, one strongly and one more modestly stained, are marked with arrows. Scale bar, 20 μm

The diminished suppression effect of light on the arousal levels and the locomotor activities in the PK2−/− mice implicates that PK2 signaling is likely involved in ipRGC-brain neural pathways, as the ipRGC have been shown as the only photic channels for the central transmission of the non-visional functions of light, including the locomotor activity suppression and sleep induction [22–25, 32, 33]. We thus examined the likely expression of PK2 in the ipRGC. As shown in Figs. 3a and 4, PK2 is quite roburstly expressed in some retinal ganglion cells. Further co-immunostaining studies indicated that all OPN4-positive retinal ganglion cells express PK2 (28/28 cells, Fig. 3). The ~100 % co-expression of PK2 and OPN4 in the ipRGC indicated that PK2 of the ipRGC projects to the SCN and other non-visional light functional areas of brain, such as the superior colliculus (SC) [34–36].

**Fig. 5:**
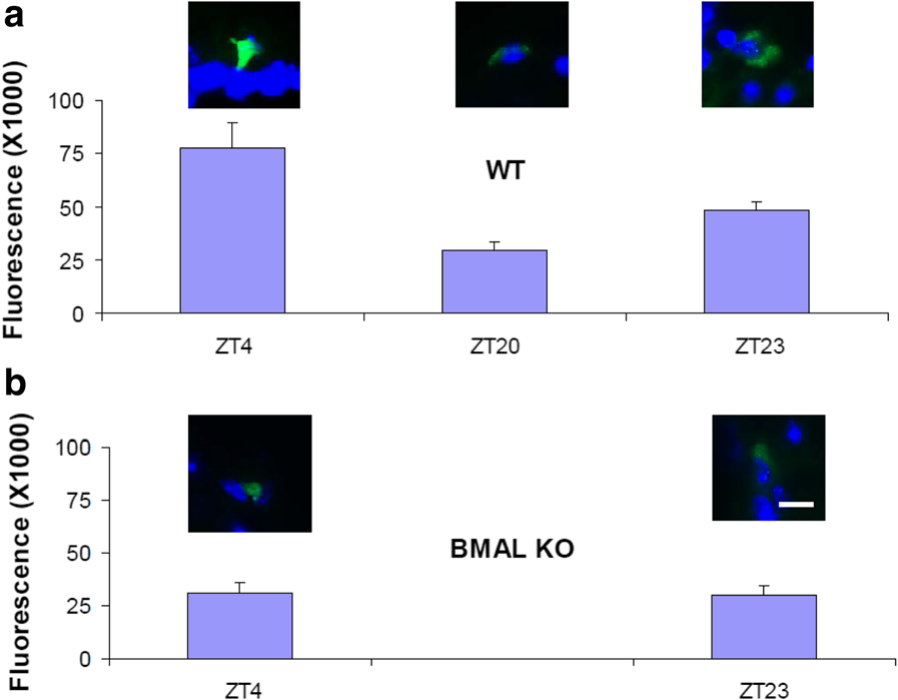
Clock-dependent oscillatory expression of PK2 in the ipRGC. The immunoflurescence intensity of PK2 was quantified and shown as mean ± SEM. The fluorescence intensity of PK2 levels in the ipRGC were oscillatory in wild type mice (**a**), but not in Bmal-deficient mice (**b**) (two way ANOVA, *P* < 0.001 genotype effect, *P* < 0.05 time effect). Peak and trough levels in the ipRGC of wild type mice were around ZT4 and ZT20, respectively. The PK2 levels in the ipRGC of Bmal1-deficient mice (BMAL KO) were consistently low, and displayed no apparent oscillation (**b**). Inserts above the columns show representative images of PK2 immunostaining of the ipRGC (green). The nuclear counterstaining is shown as blue. Bar size 10 μm

**Fig. 6:**
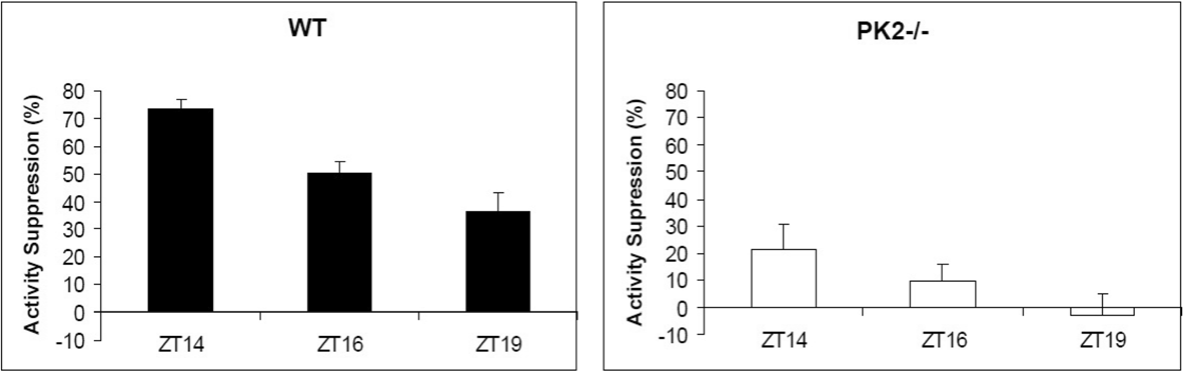
Time-dependent suppression of locomotor activities by light pulses. Light pulses (300 lux, 2.5 h) were administered to WT mice (*N* = 6) and PK2−/− mice (*N* = 6) at ZT14, ZT16 and ZT19. The percentage of activity suppression was calculated by comparing to the locomotor activities of the corresponding periods without light pulses. Light pulses significantly inhibited the locomotor activities of WT mice delivered at all three time points (*P* < 0.001, *P* < 0.001, and *P* < 0.01 for ZT14, ZT16, and ZT19, respectively, paired t-tests), but with decreasing degree of suppression (ZT14 vs ZT16, *P* < 0.001, ZT16 vs ZT19, *P* < 0.05, paired t-tests). Light pulses only significantly inhibited the locomotor activities of the PK2−/− mice when delivered at ZT14 (*P* < 0.05, paired t-test)

The PK2 expression in the ipRGC was shown to oscillate in a Bmal1-dependent manner under light and dark conditions (Fig. 5). In wild type mice, the peak and trough PK2 levels in the ipRGC were around ZT4 and ZT20, respectively. In contrast, PK2 levels in the ipRGC of Bmal1-deficient mice were constantly low, and no apparent PK2 oscillation was observed. Further, the oscillatory phase of PK2 levels in the ipRGC, including the peak and trough timing at ~ ZT4 and ~ ZT20, respectively, is quite similar to the PK2 oscillation observed in the SCN clock [6], indicating the regulation by same molecular oscillators. Consistent with the essential role of PK2 for the activity suppression by light, the PK2 expression levels in the ipRGC correlated with the extent of light suppression effect (Fig. 6). About 75 % of locomotor activity was suppressed by light pulses (300 lux) delivered at ZT14-16.5, with the suppression effect of light pulses decreasing to about 33 % when delivered at ZT19-ZT21.5, during the trough PK2 expression period in the ipRGC. Taken together, these results indicate that the oscillatory expression of PK2 in the ipRGC is a clock-dependent process.

### Differential expression of PK2 receptor in the brain targets of the intrinsically photosensitive retinal ganglion cells between the nocturnal mouse and the diurnal monkey

**Fig. 7:**
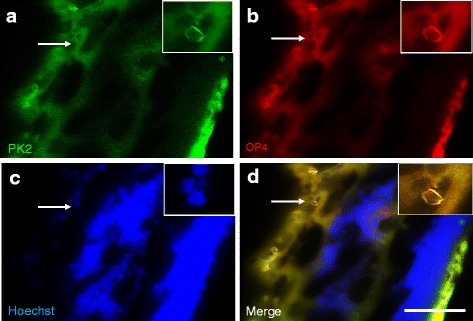
Coexpression of PK2 and OPN4 in retinal ganglion cells of the monkey retina. The PK2 immunolabeling is shown as green (**a**) whereas the OPN4 fluorescent immunolabeling is shown as red (**b**). The nuclear counterstaining is shown as blue (**c**). Coexpression of PK2 and OPN4 in the retinal ganglion cells is apparent (**d**). Inserts show the higher magnification images of representative positive cells, which are indicated by arrows. Scale bar, 20 μm

**Fig. 8:**
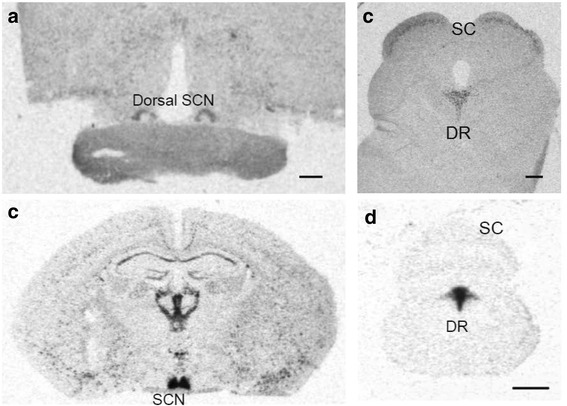
Differential expression of PKR2 mRNA in retinorecipient targets of the mouse and the monkey brains. **a**. Detection of PKR2 mRNA in the dorsal compartment of suprachiasmatic nucleus (SCN), but not in the ventral SCN, of the monkey brain. **b**. Detection of PKR2 mRNA in the entire SCN, both the ventral and dorsal compartments, in the mouse brain. **c**. Detection of PKR2 mRNA in the superficial layer of the superior colliculus (SC) of the monkey brain. **d**. Absence of PKR2 mRNA in the SC of the mouse brain. Note the expression of PKR2 in the dorsal raphe nucleus (DR) in both the mouse and monkey brains. Scale bar, 1 mm

As with the nocturnal mouse, the PK2 expression was also detected in the retinal ganglion cells of the diurnal monkey (Fig. 7a). Also identical to mice, PK2 is coexpressed with OPN4 in the ipRGC of monkeys (Fig. 7b/7d). Differential expression of PKR2, the brain PK2 receptor, in the SCN compartments was observed for the nocturnal mouse and the diurnal monkey. In the mouse brain, PKR2 is expressed in the entire SCN, covering both the ventral and dorsal compartments of the SCN (Fig. 7b) [6, 37]. It has been shown that ventral SCN is retinorecipient, i.e., receiving the retinal inputs for the light masking and circadian clock entrainment [9, 38]. The expression of PKR2 in the retinorecipient SCN in the mouse indicates that the mouse ventral SCN likely responds to PK2 signal from the ipRGC. Our previous electrophysiological studies have shown that PK2 increases the electric activities of neurons that express PKR2 [39, 40]. In the monkey brain, PKR2 is only expressed in the dorsal SCN, but is not detected in the ventral SCN (Fig. 8a). As with the nocturnal animals, the ventral SCN has been shown as the retinorecipient compartment of the SCN clock [38, 41]. The absence of the PKR2 expression in the ventral SCN indicates an inability of this branch of monkey SCN to respond to the PK2 signal from the ipRGC. Importantly, distinct expression of PKR2 between the mouse and monkey brains was also observed in the superior colliculus (SC), another critical non-vision brain target of the ipRGC [34–36]. As shown in Fig. 8c, PKR2 was robustly expressed in the superficial layer of the SC in the monkey brain. The superficial layer of the SC is known to receive inputs from the retinal ganglion cells, including the ipRGC [34–36]. In contrast, PKR2 expression was not detected in the SC of the mouse brain (Fig. 8d), indicating the absence of PK2 signaling via the ipRGC-SC in the mouse.

### Opposite effects of PK2 blockade on the arousal levels in the nocturnal mouse and the diurnal monkey

**Fig. 9:**
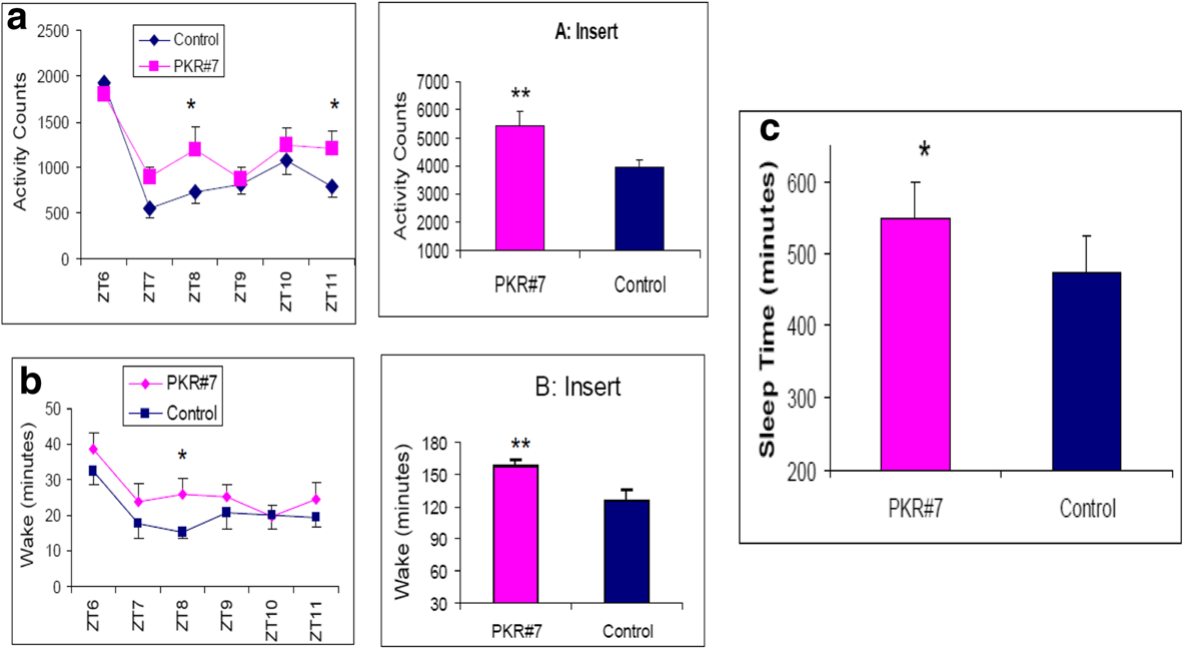
Differential effect of a PK2 antagonist PKR#7 on the locomotor activity and arousal levels in mice and monkeys. **a**. PKR#7 significantly increased the locomotor activity of the mice (*N* = 16, *P* < 0.05, Two-way ANOVA, *, *P* < 0.05, by Bonferroni’s post hoc test). **b**. PKRA83 significantly increased the wake time of the mice (*N* = 6, Two-way ANOVA, *, *P* < 0.05, by Bonferroni’s post hoc test). The inserts of A and B show the PKR#7 increased the locomotor activity and the wake minutes during the six hours day time period, respectively (**, *P* < 0.01, paired t-test). **c**. PKR#7 significantly increased the sleep time of the monkeys (*N* = 9, *, *P* < 0.05, paired t-test)

We next examined the effect of a synthetic PK2 antagonist, PKR#7, on the arousal levels in the mice and the monkeys. As shown in Fig. 9a and b, administration of PKR#7 significantly increased the locomotor activity and the wakefulness in the mice. These results are consistent with prior observation of increased wakefulness in the PK2−/− mice [31]. Thus, PK2 signal is overall inhibitory for the arousal levels for the nocturnal mice. In contrast, administration of the PK2 antagonist in the monkeys resulted in a significant increase (>70 min) of the sleep time (Fig. 9c), indicating that the PK2 signal is stimulatory for the arousal level in the diurnal monkeys.

## Discussions

**Fig. 10:**
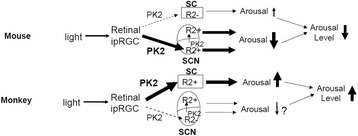
Diagram showing the differential arousal regulation by the PK2 signaling of ipRGC onto brain targets. Clock-controlled PK2 is expressed in the ipRGC, the only photic channels that transmit central non-vision functions of light. Overall, the PK2 signaling is stimulatory for the diurnal monkey and inhibitory for the nocturnal mouse, as shown by the antagonist blockade and PK2-deficiency. The differential expression of PKR2 in the retinorecipient ventral SCN and the superficial layer of SC indicates that the PK2 signaling of the ipRGC dominantly funnels through ipRGC-SCN and ipRGC-SC for the mouse and the monkey, respectively. For the nocturnal animals, the arousal stimulation via the ipRGC-SC pathway by light is minor (transient, not sustained), consistent with the absence of PKR2 in the SC of the mouse brain. The PK2 signaling of the ipRGC-SCN pathway is clearly inhibitory for the nocturnal mouse, although it is unclear whether it is inhibitory or stimulatory for the diurnal animals. SC may mediate the light-driven arousal via the ascending projections to cortices that are routed through the lateral posterior/Pulvinar complex of the thalamus. Alternatively, SC may promote arousal via the descending projections to the mesencephalic reticular formation, an important component of the ascending activation system (ref 42–45). Our model indicates that the mammalian diurnal/nocturnal determination is mediated by the differential signaling of the ipRGC onto their brain targets, and thus divergent signaling mechanisms downstream of the SCN may not be necessary. In diurnal animals, upstream clocks such as the ones in the ipRGC likely play more dominant roles than the SCN, at least for arousal regulation under light and dark condition

In the current study, we have shown that PK2 is expressed in the centrally projecting ipRGC. As the ipRGC are the only photic channels for the central transmission of the non-visional functions of light, the absence of the sustained light-induced activity suppression and sleep induction in the PK2−/− mice indicates that the PK2 signaling of the ipRGC is critical for mediating the direct effect of light on the arousal regulation in the nocturnal mouse. The Bmal1-dependent oscillation of PK2 in the ipRGC indicates that PK2 expression in the ipRGC is controlled by the known circadian oscillator. Blocking the PK2 signaling via administration of a PK2 antagonist demonstrated the opposite effects of the PK2 signal on the arousal levels: being inhibitory for the nocturnal mouse and stimulatory for the diurnal monkey. Between the nocturnal mouse and the diurnal monkey, PK2 receptor is differentially expressed in the retinorecipient SCN and SC, brain projection targets of the ipRGC. Taken together, these results indicate a likely mechanism of the nocturnal and diurnal divergence lies in the differential PK2 signaling of the ipRGC onto their brain targets (Fig. 10).

The PK2 signaling of the ipRGC likely regulates the arousal levels via impinging on the brain targets of the ipRGC, particularly the SCN and the SC. The differential expression of the PK2 receptor (PKR2) in the retinorecipient compartment of the SCN and the retinorecipient superficial layer of the SC may then underlie the opposite effects of light on the arousal levels between the nocturnal mouse and the diurnal monkey. In the mouse brain, PKR2 is robustly expressed in the retinorecipient SCN, but absent in the SC, and the PK2 signaling of the ipRGC thus funnels through arousal-inhibitory ipRGC-SCN pathway in the nocturnal mouse. In contrast, PKR2 is not expressed in the retinorecipient ventral SCN of the monkey brain, but strongly expressed in the retinorecipient superficial layer of the SC, and thus the ipRGC-SC pathway dominates in the diurnal monkey.

The PK2 signaling of the ipRGC-SC pathway is likely to be stimulatory for the arousal levels. SC has previously been indicated as a critical nucleus for the light-induced arousal or other higher brain functions, such as attention, that are closely tied with increased arousal [42–45]. In the monkeys, bilateral lesions of the SC have been found to drastically affect the arousal levels, including response to light [42]. Lesion studies in rats have revealed that the SC is required for EEG desynchronization (arousal) in response to light flashes [43]. This rat lesion study suggests that the ipRGC-SC pathway may be stimulatory for the arousal levels for the nocturnal animals, at least briefly in response to light flashes. Over a longer duration, light is inhibitory for the arousal levels of the nocturnal animals as the inhibitory ipRGC-SCN pathway dominates. In diurnal mammals such as monkeys, SC may mediate the light-driven arousal via the ascending projections to cortices that are routed through the lateral posterior/Pulvinar complex of the thalamus [46, 47]. The lateral posterior/Pulvinar complex of the thalamus is known to play a critical role for higher function such as attention [48]. In this regard, wakefulness may be viewed as low level attention. It is well known that, compared to the nocturnal animals, the overall size of the SC, the lateral posterior/Pulvinar complex of the thalamus, and the associated cortices are all significantly enlarged and expanded in the diurnal mammalian species, such as the primates [49, 50]. As the mammalian species are believed to start being nocturnal [51], diurnality of the mammals may evolve via the expansion of the arousal-stimulatory ipRGC-SC pathway and the simultaneous diminishment of the arousal-inhibitory ipRGC-SCN pathway. Our model (Fig. 10) indicates that the nocturnal/diurnal determination of arousal levels lies in the upstream of the SCN circadian clock, and divergent signaling downstream of the SCN clock may not be necessary. The melatonin rhythms, known to lie downstream of the SCN clock, will operate in the same phase of the SCN clocks and thus no difference will be exhibited in the nocturnal and diurnal mammals. Our model also argues against the previously presumed central role of SCN as the master clock for diurnal mammals, such as monkeys, at least for the regulation of arousal levels under light and dark conditions. Although the supporting evidence for the SCN as the master circadian clock is overwhelmingly strong for the nocturnal mammals, such claim has actually limited supporting evidence in the case of the diurnal animals [8]. The well cited work of increased sleep by the SCN lesion in squirrel monkeys [52], interpreted as the arousal-promoting of the SCN for the diurnal animals, could be due to the concurrent lesions to the retinohypothalamic tract, which would damage projections to both SCN and SC. Under light and dark condition, it is likely that the ipRGC play central roles for the regulation of arousal levels regardless whether the mammals are diurnal or nocturnal. In nocturnal mammals, ipRGC and SCN act in sequential neural projections (and with the similar oscillatory phase) to regulate arousal levels, thus nocturnal activity patterns are displayed. In diurnal mammals, the arousal-stimulatory ipRGC-SC projections overcome the diminished arousal-inhibitory ipRGC-SCN projections, and thus diurnal activity patterns are exhibited

## Methods

### Animals

PK2−/− mice and their littermate wild type controls in mixed genetic background were generated as described [29, 31]. Bmal1−/− mice were produced by crossing from heterozygous mice that were procured from Jackson laboratory. Mice were fed at libo and housed at regular light/dark cycle, with lights (~150 lux white light) on at 7:00 a.m. (Zeitgeber Time ZT0, light period ZT0-ZT12) and lights off at 7:00 p.m. (ZT12, dark period ZT12-ZT0). All animal procedures were approved by appropriate institutional animal use committee.

### Measurement and analysis of the locomotor activity in mice

Monitoring of the locomotor activity was carried out as described [29]. Briefly, mice were individually housed with cages equipped with infrared beams for the monitoring of the locomotor activity (AccuScan Instrument Inc. Columbus, OH). Mice were housed at regular 12 h Light (~150 lux white light): 12 h Dark cycle. The locomotor activities were recorded as counts per 10-min interval and were analyzed in 30 or 60 min pins. Light pulses or dim light at the indicated intensities were administered.

### Measurement and analysis of the arousal level in mice

Electrodes for recording the electroencephalographic (EEG) and electromyogram (EMG) signals were implanted as described [29, 31]. The mice were connected to a swivel system of tether/commutator system (Plastics One, Roanoke, VA) for the collection of the EEG/EMG signals. The EEG/EMG signals were amplified using a Grass Model 78 (Grass Instruments, West Warwick, RI) and filtered (EEG: 0.3–100 Hz, EMG: 30–300 Hz) before being digitized at a sampling rate of 128 Hz, stored on a computer. After sleep data were collected, EEG/EMG records were scored with SleepSign software sleep scoring system (Kissei Comtec America, Irvine, CA) as described [31]. Mice were housed at a regular 12 h light/12 h dark cycle. Light pulses or dim light at the indicated intensities were administered.

### *In situ* hybridization

Procedures for *In situ* hybridization were carried out similarly as described [6, 7]. Tissue sections were cut at −20 °C, and then fixed with 4 % paraformaldehyde, followed by three washes of 0.1 M phosphate buffer, air-dried, and stored at −20 °C until use. For *In situ* hybridization, sections were dried at room temperature, followed by pretreatment of proteinase K (1 μg/ml). Sections were then air-dried and hybridized with S [35]-labelled riboprobes by incubation at 60 °C for 18 h. After hybridization, tissue sections were treated with RNAase (20 μg/ml) (Sigma-Aldrich, St. Louis, MO), decreasing salinity washes and high stringency (68 °C) wash. After dehydration and air-drying, tissue sections were exposed to Kodak Biomax film. Images were captured with image analysis system (MCID, Imaging Research, Ontario, Canada).

### Immunohistochemistry

Immunohistochemistry was performed according to previous publications [53, 54]. Retinal sections were mounted onto coated glass slides. Sections were rehydrated in PBS for 20 min then immersed in a blocking buffer containing 2 % BSA, 0.5 % Tween-20 and 0.05 % Triton-X 100 for 1 h. Primary antibody for PK2 (Hamster monoclonal, 1:200, Roche Inc.) or OPN4 (Affinity purified rabbit polyclonal, 1:200, Millipore Inc.) was added to the sections overnight at 4 °C. Slides were washed with PBS containing 0.5 % Tween-20 five times for 5 min each. Anti-rabbit or anti-hamster secondary antibodies (Alexa Fluor 488 or 555 1:2000; Invitrogen Inc.) were then applied, followed by incubation with 10 μg/ml Hoechst 33342 (Invitrogen Inc) for 5 min at room temperature to stain the nucleus. Sections were viewed under a Nikon inverted fluorescence microscope (Model TE-2000U; Nikon Inc, Tokyo, Japan). Images were captured with a SPOT digital camera (Diagnostic Instruments, Inc, Sterling Heights, MI). Immunofluorescence intensity was quantified with Image J. For DAB (3,3′-diaminobenzidine) immunostaining, sections were incubated with anti-PK2 antibody (Hamster monoclonal, 1:500 dilution) antibody, followed by incubation with biotinylated anti-hamster secondary antibody. Color development of DAB immunostaining was carried out with the standard ABC method [52].

### Pharmacological experiments of examining the effect of a PK2 antagonist on the activity or arousal levels in the mice and the monkeys

A PK2 antagonist (PKR#7) was prepared similarly as described [55]. PKR#7 (40 mg/kg) was administered to the mice intraperitoneally at ZT6. PKR#7 (10 mg/kg) was administered to the monkeys intramuscularly at ZT10. For the pharmacological experiments, animals were treated with either the vehicle or antagonist and then crossed over with the opposite treatments 1 week later to form paired controls.

Sleep and activity data of the PK2 antagonist or control-treated mice were acquired and analyzed as described for the PK2−/− mice. For the sleep studies of the monkeys, young adult monkeys (Macaca fascicularis) were housed under standard light (white light ~250 lux) and dark cycle. The measurement and analysis of the arousal levels in the monkey were carried out as follows. A wearable wireless sleep tracker, similar to described previously for human subjects [56–59] and for non-human primates [60], was used. This wireless system enabled remote monitoring of the sleep/wake status of the monkeys for an ambulatory setting for a long time with minimal disturbing of the monkeys. The sleep data obtained from the wireless sleep tracker were verified with with concurrent recording of infrared video camera. The sleep data of the sleep trackers were retrieved daily with mobile phones that were seated about ten meters away from the animal cages, without physical contact with the monkeys. Previous studies have shown excellent agreement of sleep data obtained by the sleep tracker, video camera and classical sleep/wake data obtained by the EEG/EMG method [56, 59, 61].

### Statistics

To reduce the impact of data variations due to ultradian rhythms, the measurements of mouse locomotor activity and EEG/EMG were performed two times that were separated by 3 or 4 days, and the average values of these two measurements were used in statistical analyses. Statistical analyses were performed with 1 or 2 ways ANOVA by using GraphPad Prism Software Version 5.0 (San Diego, CA), followed by appropriate post tests.

## Abbreviations

EEG, electroencephalography; EMG: electromyogram; ipRGC, intrinsically photosensitive retinal ganglion cells; LD, light/dark cycle; OPN4, melanopsin; PK2, prokineticin 2; RHT, retinohypothalamic tract; SC, superior colliculus; SCN, suprachiasmatic nucleus; ZT, Zeitgeber Time.
